# Ecological Service Value Tradeoffs: An Ecological Water Replenishment Model for the Jilin Momoge National Nature Reserve, China

**DOI:** 10.3390/ijerph19063263

**Published:** 2022-03-10

**Authors:** Jin Huang, Hao Yang, Wei He, Yu Li

**Affiliations:** 1 Chemistry Experiment Teaching Center, College of Materials Science and Chemical Engineering, Harbin Engineering University, Harbin 150001, China; 2 MOE Key Laboratory of Resources Environmental Systems Optimization, College of Environmental Science and Engineering, North China Electric Power University, Beijing 102206, China; 120212232080@ncepu.edu.cn (H.Y.); 120202232011@ncepu.edu.cn (W.H.)

**Keywords:** interval two-stage stochastic programming (ITSP) method, ecosystem function restoration, ecological water replenishment, ecological service value, carbon sink

## Abstract

Wetlands as an important ecosystem type have been damaged in recent years and restoration of wetland ecosystem functions through ecological water replenishment is one of the important ways. The present study involved the construction of a novel ecological water replenishment model for Jilin Momoge National Nature Reserve (JMNNR) using the interval two-stage stochastic programming (ITSP) method. Breaking down traditional economic models that often sacrifice environmental benefits, the model aims to replenish the ecological water in JMNNR, allocate the ecological water resources scientifically, restore the wetland function of the reserve, improve the functional area of the reserve, enhance the net carbon sequestration capacity of the reserve, and complete the reconstruction of the ecosystem, while considering the ecological service value (ESV) of the reserve to achieve a joint increase in the ecological and economic benefits. The ITSP model constructed in the present study overcame the limitation that the original project recommendation was a single recommended value, and the results are presented in the form of intervals to improve flexibility in decision making to allow the individuals responsible for under-taking decisions to bring focused adjustments according to the actual decision-making conditions and increase the selectivity of the decision-making scheme. The present report discusses the construction of an ITSP model for the ecological water replenishment of JMNNR in an attempt to effectively improve both economic benefits and ecosystem restoration of the reserve, achieve the reconstruction of the JMNNR ecosystem, and provide a selective decision space for the key decision-makers to formulate and optimize the project operation and the management plan. The use of the ITSP model as a pre-procedural basis for the implementation of the project and the simulation of the effects of the implementation of the project can effectively avoid the decision limitations that exist when carrying out the project directly. The ITSP model constructed in this paper can also be used as a theoretical guide for water replenishment projects in different areas of the world, and the model parameters can be reasonably adjusted to achieve better results when used according to the actual local conditions.

## 1. Introduction

A wetland is a transitional zone of biodiversity and productivity at the interface of land and water. This zone is characterized by shallow water covering wet soil and scattered vegetation, either submerged or emerging [1]. Wetlands are considered one of the most productive and economically valuable ecosystems globally [2] as they offer several important ecosystem services to humans, such as carbon storage, water purification, flood control, biodiversity conservation, and cultural entertainment [3,4,5,6]. However, rapid population growth and economic growth, combined with long-term overdevelopment, have resulted in the overwhelming of the world’s wetland ecosystems, pushing several of them to the brink of collapse [7]. Currently, over 50% of the wetlands in the world have already been lost. This phenomenon is even further critical in China, a country with over 60% of its wetlands damaged to varying degrees [8]. When the hydrological environment is damaged due to natural disturbance or anthropological factors, it leads to diminished biological habitats and reduced water sources, which challenge the survival of biological species and even impact the structure of different biological communities, thereby destroying biological diversity. This ultimately changes the structure of the wetland ecosystems and destroys their stability [9,10]. Therefore, it is imperative to focus on wetland restoration and maintenance of the wetland ecosystem functions. Jilin Momoge National Nature Reserve (JMNNR) is located in northeastern China and is a typical wetland type reserve and internationally important wetland. In recent years, the wetland area of the JMNNR has also gradually shrunk due to factors such as climatic drought, and the quality has continued to decline, with the ecological environment deteriorating. It is imperative that measures are taken to continuously restore wetlands.

The ecological restoration of wetlands mainly encounters two major issues: one is the sustainable availability of regional water resources, which has been impacted by the prevailing global water scarcity [11], and the other is the lack of efficient methods and technologies for the utilization of regional water resources for wetland restoration [12]. Wetland ecological water replenishment is one of the most effective and direct methods available for wetland area restoration and wetland ecosystem improvement [13,14]. Ecological recharge has been used widely in wetland restoration with demonstrated good results. Yang et al. [15] developed an integrated economic-hydrological model to examine the cost-effectiveness and restoration of a 75 km^2^ wetland restoration scenario in the Southern Tobacco Creek watershed in the Canadian Prairies, and the simulations found the importance of spatially directed wetland restoration based on the economic cost to environmental benefit ratios to achieve cost effectiveness; Hua et al. [16] proposed an ecohydrological method for determining the potential area of freshwater wetlands to be restored, establishing a multi-objective habitat suitability index model modification area to be restored, and restoring the balance between the hydrological network and the ecological water demand and ecological water supply modification areas; Chen et al. [17] used long time series remote sensing data to improve hydrological conditions in the Zhalong wetland by ecological water replenishment project. These scholars have used hydrological models to restore wetlands in the study area by means of ecological water replenishment and have achieved some results. However, the values obtained from these simulations are all single definite values and do not take into account the uncertainties associated with the restoration of wetland ecosystems by means of ecological water replenishment. For example, the amount of water replenishment is usually used to estimate the area of wetland that can be restored, but the cross-section of the wetland is not a regular rectangle, which leads to uncertainty in the area of restored wetland. At the same time, the restoration effect in the form of a single fixed value is not sufficient to cope with the various uncertainties during the implementation of the project, which brings limitations to the implementation of the project. Therefore, it is necessary to take into account the uncertainties in the wetland ecological water replenishment project to facilitate the development and implementation of the project.

ITSP methods have been widely used to solve uncertainty problems in a variety of research fields. Luo et al. [18] constructed an ITSP model based on simulation to control non-point source agricultural pollution and determined an optimal land retirement scheme to control this pollution by minimizing the long-term economic and environmental costs. Fu et al. [19] combined the water rights trading model with the interval-two-stage parameter stochastic programming model, and then applied the resulting model to multi-regional, multi-source, and multi-use water users in Sanjiang Plain to simulate each user’s optimal committed water consumption in this region. Maqsood et al. [20] applied the ITSP method to a solid waste management system under uncertain conditions and developed the waste logistics operation mode with minimum system cost and maximum system feasibility. Therefore, the application of the ITSP method to wetland ecological water replenishment projects can effectively solve the uncertainty in the implementation of the project.

Two model approaches have been used to restore the suitable habitat area for rare bird species by Liao et al. [21,22]. However, the constructed model was focused only on the effect of recharge on the migration of water birds while not considering the economic benefits of the reserve and the restoration of the ecological functions of this wetland region.

Therefore, to improve the water scarcity scenario in the lake wetlands of JMNNR, the present study, in cooperation with the western Jilin water supply project, constructed a novel ecological water replenishment model based on the ITSP method for JMNNR, to maximize the economic benefits for the ecological replenishment of each lake. Breaking the limitations of traditional economic models, completely considering the ecological functions of the reserve, restoring the functional area of each lake wetland, and enhancing the ESV of each ecological service index, such that both economic benefits and ecological function of the wetland are optimized.

## 2. Study Area

**Figure 1:**
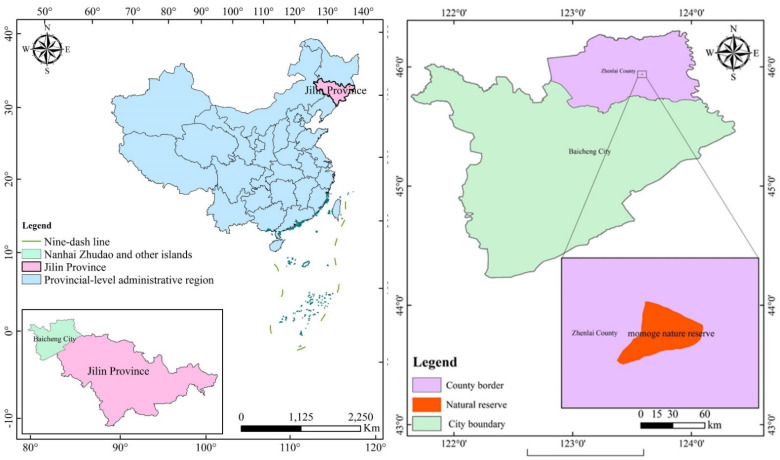
The geographical location of Jilin Momoge National Nature Reserve.

The Jilin Momoge National Nature Reserve is known as “the kidney of western Jilin.” JMNNR is situated at the confluence of two rivers, the Nenjiang and the Taoer, in Zhenlai County, Baicheng City, Jilin Province. Wukeshu and Hatuqi in Zhenlai County border the region to the northwest [23]. The geographical location of JMNNR is depicted in Figure 1. JMNNR receives annual precipitation of less than 400 mm, which is concentrated mainly between the months of June and August. Since the natural precipitation in this region is inadequate and unevenly distributed across the year, and the annual evaporation reaches up to 900–1000 mm, which is much higher than the annual precipitation, there is a serious shortage of water resources in the Momoge Reserve [24]. Moreover, the wetland area has reduced over time due to the action of anthropological factors, such as reclamation of wetlands in the protected regions, which have resulted in the withering of vegetation, migration of birds, environmental degradation, and loss of proper functioning of the ecosystem [25,26]. In order to restore the wetland area and the ecosystem function of JMNNR, it was decided that the Jilin Western Water Supply Project would be utilized as a water supply source for the ecological replenishment of the wetland (the Etoupao, Haernaopao, and Yuanbaotupao lakes in the reserve are classified as important from the perspective of ecological protection by the Jilin Western Water Supply Project) and that the water resources would be reasonably allocated for reducing the ecological deterioration of the JMNNR and the surrounding regions. The design objective was to restore the wetlands of the JMNNR to the levels of the 1950s and 1960s and recreate the natural scenery of the wetlands with fertile grass, pleasant water, fragrant flowers, and songbirds.

## 3. Model Construction

### 3.1. Overview of ITSP Methods

In this paper, the uncertainty of variables is expressed in the form of interval, and the ITSP model is constructed as follows:

(1) $$Maxf^{\pm }=C^{\pm }\cdot X^{\pm }$$

subject to:

(2) $$A^{\pm }\cdot X^{\pm }\geq B^{\pm }$$

(3) $$X^{\pm }\geq 0$$

where, $A^{\pm }=\left[A^{-},A^{+}\right]\in {\left\{R^{\pm }\right\}}^{m\times n}$, $B^{\pm }=\left[B^{-},B^{+}\right]\in {\left\{R^{\pm }\right\}}^{m\times 1}$, $C^{\pm }=\left[C^{-},C^{+}\right]\in {\left\{R^{\pm }\right\}}^{1\times n}$, $X^{\pm }=\left[X^{-},X^{+}\right]\in {\left\{R^{\pm }\right\}}^{n\times 1}$.

### 3.2. Construction of Optimised Ecological Water Supply Model Based on ITSP Method

The optimization objective was chosen to maximize the economic benefits of JMNNR, while both ecological and environmental benefits were considered. The study used the ITSP method to construct an ecological water replenishment model for JMNNR, taking into account the disposable water volume, lake planning scope, ecological benefit, net carbon sequestration, and other factors as constraints. The study area included 18 lakes located in JMNNR, and the corresponding four water intake projects were used for optimizing the initial scheme for ecological water replenishment under dry, flat, and abundant water years to achieve the maximum economic benefit, restore the functional area of the wetland, and reconstruct the Momoge wetland ecosystem.

The objective function of the ITSP model constructed in the present study was as follows:

(4) $$Maxf^{\pm }=\sum_{i=1}^{18}\sum_{j=1}^{4}\sum_{h=1}^{3}\sum_{k=1}^{19}Y_{ij}^{\pm }\cdot ESV_{jk}^{\pm }\cdot FA_{ijh}^{\pm }$$

where $f^{\pm }$ denotes the total economic benefits obtained from JMNNR, i indicates the 18 lakes in the Momoge National Nature Reserve; j=1,2,3,4 indicates four different ecological functional areas, namely, fish ponds, crab ponds, reed wetlands, and marsh wetlands, respectively; h=1,2,3 represents the flood flow scenarios, namely, dry, flat, and abundant water years, respectively; $P_{h}$ denotes the probability of different traffic scenarios; k=1,2…19 indicates the ecosystem service function of Momoge Reserve; $OTEB_{kh}^{\pm }$ denotes the ecological benefit of the ecological service function k of the water replenishment optimization scheme under different flow scenarios (10^6^ yuan); $Y_{ij}^{\pm }$ represents the 0–1 variables; $ESV_{jk}^{\pm }$ denotes the ecological benefits per unit area of ecological service function k for different regions (10^6^ yuan/10^3^ hm^2^), and $FA_{ijh}^{\pm }$ denotes the different ecological function areas of each lake under different flow scenarios (10^3^ hm^2^).

#### 3.2.1. Minimum Water Constraint

The replenishment of each lake bubble needs to meet the minimum requirements of each ecological function area [21]:

(5) $$QT_{i}^{\pm }-QS_{ih}^{\pm }\geq \sum_{j=1}^{4}A_{ij\min}^{\pm }\cdot QR_{ij}^{\pm },\forall i,h$$

where $QT_{i}^{\pm }$ denotes the ecological replenishment in each lake (10^4^ m^3^); $QS_{ih}^{\pm }$ denotes the amount of water deficit under different scenarios (10^4^ m^3^); $A_{ij\min}^{\pm }$ denotes the minimum area requirement in the different ecological functions of the lake i (10^3^ hm^2^), and $QR_{ij}^{\pm }$ denotes the ecological water demand per unit for different ecological function zones of each lake (10^4^ m^3^/10^3^ hm^2^).

#### 3.2.2. Water Supply Capacity Constraints

The water supply capacity of the four water supply outlets in the study area:

(6) $$QT_{i}^{\pm }-QS_{ih}^{\pm }\leq QID_{ih}^{\pm }+QND_{ih}^{\pm }+QFD_{ih}^{\pm }-QL_{i}^{\pm },\forall i,h$$

(7) $$\sum_{i=1}^{9}QFD_{ih}^{\pm }\leq QTF_{nh}^{\pm },n=1,\forall h$$

(8) $$\sum_{i=10}^{12}QFD_{ih}^{\pm }\leq QTF_{nh}^{\pm },n=2,\forall h$$

(9) $$\sum_{i=13}^{16}QFD_{ih}^{\pm }\leq QTF_{nh}^{\pm },n=3,\forall h$$

(10) $$\sum_{i=17}^{18}QFD_{ih}^{\pm }\leq QTF_{nh}^{\pm },n=4,\forall h$$

(11) $$QT_{i}^{\pm }\leq QAD_{i}^{\pm },\forall i$$

(12) $$QID_{ih}^{\pm }\leq QI_{i}^{\pm },\forall i,h$$

(13) $$QND_{ih}^{\pm }\leq QN_{i}^{\pm },\forall i,h$$

where $QID_{ih}^{\pm }$, $QND_{ih}^{\pm }$, and $QFD_{ih}^{\pm }$ denote the local water supply, normal water supply, and floodwater supply, respectively, under different flow scenarios in each lake (10^4^ m^3^); $QL_{i}^{\pm }$ denotes the amount of water lost during transmission (10^4^ m^3^); n=1,2,3,4 indicates the four water intakes in the study area represented by Sanfengan of Baisha Irrigation District, Zhushan pumping station, Shijianfang intake sluice, and Haernao pumping station, respectively; $QTF_{nh}^{\pm }$ denotes the total flood resource at the intake for different scenarios (10^4^ m^3^); $QAD_{i}^{\pm }$ denotes the maximum amount of water replenished in each lake (10^4^ m^3^); $QI_{i}^{\pm }$ and $QN_{i}^{\pm }$ denote the local water supply and normal water supply, respectively (10^4^ m^3^).

#### 3.2.3. Water Replenishment Sequence Constraints

Fish and crab ponds are given priority over reed and marsh wetland recharge in the recharge project, and reed wetlands are given priority over marsh wetland recharge.

(14) $$\sum_{j=3}^{4}FA_{ijh}^{\pm }\cdot QR_{ij}^{\pm }=\left\{ \begin{array}{l} QID_{ih}^{\pm }+QND_{ih}^{\pm }+QFD_{ih}^{\pm }-QL_{i}^{\pm }-\sum_{j=1}^{2}FA_{ijh}^{\pm }\cdot QR_{ij}^{\pm },\\ if\sum_{j=1}^{2}FA_{ijh}^{\pm }\geq \sum_{j=1}^{2}A_{ij\min}^{\pm }\\ 0\;,\;if\sum_{j=1}^{2}FA_{ijh}^{\pm }\leq \sum_{j=1}^{2}A_{ij\min}^{\pm }\; \end{array}\right.,\forall i,h$$

(15) $$FA_{i4h}^{\pm }\cdot QR_{i4}^{\pm }=\left\{ \begin{array}{l} \sum_{j=3}^{4}FA_{ijh}^{\pm }\cdot QR_{ij}^{\pm }-FA_{i3h}^{\pm }\cdot QR_{i3}^{\pm },if\;FA_{i3h}^{\pm }\geq A_{ij\min}^{\pm }\\ 0\;,\;if\;FA_{i3h}^{\pm }\leq A_{ij\min}^{\pm }\; \end{array}\right.,\forall i,h$$

#### 3.2.4. Functional Zone Area Constraints

The area of each functional area must not be higher than the maximum area of that area [21]:

(16) $$\sum_{j=1}^{4}FA_{ijh}^{\pm }\leq TFA_{i}^{\pm },\forall i,h$$

(17) $$FA_{ijh}^{\pm }\leq A_{ij\max}^{\pm },\forall i,j,h$$

(18) $$\sum_{j=1}^{4}FA_{ijh}^{\pm }\geq C_{i}^{\pm }\cdot (QT_{i}^{\pm }-QS_{ih}^{\pm }),\forall i,h$$

(19) $$\sum_{j=1}^{4}FA_{ijh}^{\pm }\cdot QR_{ij}^{\pm }=QT_{i}^{\pm }-QS_{ih}^{\pm },\forall i,h$$

where $TFA_{i}^{\pm }$ is the upper limit of the area of each lake planning area (10^4^ m^3^) and $A_{ij\max}^{\pm }$ is the upper limit of the area of the different ecological function zones of the lake i (10^4^ m^3^).

#### 3.2.5. Eco-Efficiency Constraints 

The value of ecological services generated in different traffic contexts [21]:

(20) $$OTEB_{kh}^{\pm }=\sum_{i=1}^{18}\sum_{j=1}^{4}FA_{ijh}^{\pm }\cdot Y_{ij}^{\pm }\cdot ESV_{jk}^{\pm },\forall k,h$$

(21) $$OTEB_{kh}^{\pm }\geq TEB_{k}^{\pm },\forall k,h$$

(22) $$\begin{array}{c} \sum_{i=1}^{18}\sum_{j=1}^{4}FA_{ijh}^{\pm }\cdot Y_{ij}^{\pm }\cdot \sum_{k=1}^{19}ESV_{kh}^{\pm }-\sum_{i=1}^{18}(QT_{i}^{\pm }-QS_{ih}^{\pm }-QFD_{ih}^{\pm })\cdot 0.18\\ -\sum_{i=1}^{18}QFD_{ih}^{\pm }\cdot 0.11\geq SEB_{m}^{\pm },\;\forall h \end{array}$$

where $OTEB_{kh}^{\pm }$ denotes the ecological benefits of the recharge optimization scheme for the ecological service function k under different flow scenarios (10^6^ yuan); $TEB_{k}^{\pm }$ denotes the ecological benefit of the ecological service function k generated by the recommended scheme of the project (10^6^ yuan); and $SEB_{m}^{\pm }$ denotes the total ecological benefit generated by the original scheme (10^6^ yuan).

#### 3.2.6. Net Carbon Sequestration Constraint 

Carbon sink capacity of ecological functional areas [22]:

(23) $$OTCS_{ih}^{\pm }=\sum_{j=1}^{4}FA_{ijh}^{\pm }\cdot Y_{ij}^{\pm }\cdot NCSA_{ij}^{\pm },\forall i,h$$

(24) $$OTCS_{ih}^{\pm }\geq TCS_{i}^{\pm },\forall i,h$$

where $OTCS_{ij}^{\pm }$ denotes the NSC of the ecological function area j for different regions after optimization (t); $NCSA_{ij}^{\pm }$ denotes the NSC capacity of the ecological function area j of the lake i (t/10^3^ hm^2^); and $TCS_{ij}^{\pm }$ denotes the NSC in the ecological function area j for different regions (t).

#### 3.2.7. Non-Negative Constraints

The optimized solution represents a non-negative replenishment of each lake.

(25) $$QS_{ih}^{\pm },QFD_{ih}^{\pm },FA_{ijh}^{\pm },QID_{ih}^{\pm },QND_{ih}^{\pm },QFD_{ih}^{\pm }\geq 0,\forall i,j,h$$

## 4. Results and Discussion

The current study entailed developing an ecological replenishment model for JMNNR using the ITSP method via the ecological replenishment route in order to maximize economic benefits and restore the functional area of the JMNNR through ecological replenishment projects. The aim was to alleviate the water scarcity issue in the lake wetlands of this reserve while increasing the NSC capacity and ensuring the ESV. The application of the model achieved the restoration of the function of the lake-lake wetland and the reconstruction of the ecosystem.

### 4.1. Economic Index Analysis of the Ecological Water Replenishment Project in JMNNR Using the ITSP Method

#### 4.1.1. Economic Benefit Analysis of the Ecological Water Replenishment Project in JMNNR Using the ITSP Method

Economic benefits are an important manifestation of regional economic development. Therefore, the ecological replenishment model constructed in the present study using the ITSP approach, while aimed at restoring the wetland functions in JMNNR, also ensured the maximization of the local economic benefits. According to the simulation results of the ITSP model, the economic benefits of JMNNR were [8128.86, 13,222.55] × 10^4^ Yuan. The model simulation results are presented as interval values to facilitate setting practical decision options according to the actual situation. The ESV generated varies with the type of restoration area, which requires the decision-makers to plan and select the most appropriate decision option.

#### 4.1.2. Analysis of the ESV of the Ecological Water Replenishment Project in JMNNR Using the ITSP Method

JMNNR’s ecological water replenishment project considers the project’s benefits while reconstructing the wetland ecosystem and restoring wetland functions. The project benefits are mainly reflected in the restoration of the wetland areas and functions and the value of the ecosystem services, where the latter refers to the benefits gained by maintaining the ecosystem functions of the Earth’s life support systems. The recommended project solutions and the ITSP method optimization results for the ESV under different index systems in JMNNR are presented in Table 1.

Nineteen types of ecological service functions were analyzed in the present study, which mainly generated ESVs in the following order: pollution absorption capacity ((435.99, 761.75) × 10^6^ yuan) > flood storage ((349.78, 536.25) × 10^6^ yuan) > plant adsorption ((301.15, 526.16) × 10^6^ yuan) > cooling and humidification ((297.40, 515.23) × 10^6^ yuan) > conservation of rare waterfowl ((284.30, 474.92) × 10^6^ yuan) > soil conservation ((252.38, 431.80) × 10^6^ yuan) > crab ((187.97, 270.20) × 10^6^ yuan) > leisure and tourism ((106.02, 177.10) × 106 yuan) > water supply ((100.45, 144.40) × 10^6^ yuan) > ecological habitat ((70.84, 118.33) × 10^6^ yuan) > oxygen release ((67.62, 115.31) × 10^6^ yuan) > fish ((63.10, 90.71) × 10^6^ yuan) > cityscape ((58.00, 96.89) × 10^6^ yuan) > Scirpus triqueter ((24.78, 44.12) × 10^6^ yuan) > reeds ((24.78, 41.73) × 10^6^ yuan) > natural landscapes ((23.30, 38.91) × 10^6^ yuan) > nutrient circulation ((19.76, 33.84) × 10^6^ yuan) > research and education ((14.74, 24.62) × 10^6^ yuan) > vegetation carbon sequestration ((7.34, 12.51) × 10^6^ yuan) (in flat water years). The indicators ranked first, third, fifth, and sixth were pollution absorption capacity, plant sorption, conservation of rare waterfowl, and soil conservation, respectively. The indicators ranking second and fourth were flood storage and cooling and humidification, respectively, which are also regulatory services. The rankings demonstrate that the ITSP model constructed in the present study focused on the support and regulation of the ecological functions in JMNNR while not focusing on the pursuit of economic gains and primarily considering only the reconstruction and maintenance of ecological functions in the reserve. The ESV increased sequentially in the dry, flat, and high water years as the water period progressed. This could be attributed to the low level of available water resources and the lower ESV generated in dry years, whereas the high level of available water resources and greater flexibility in water allocation in high water years increased the ESV generated by the amount of water.

**Figure 2:**
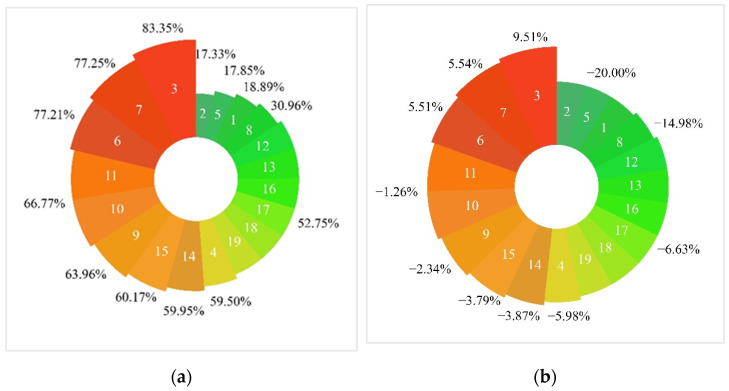
Change in the ESV based on the ITSP model in JMNNR compared to the project recommendation. (**a**) Upper limit of the change in ESV from the project recommendation. (**b**) Lower limit of the change in ESV from the project recommendation.

The changes in the ESVs compared to the project recommendations for JMNNR after simulation and optimization using the ITSP model are presented in Figure 2. The ESV compared to the project recommendation was the highest for reed, reaching a value of up to (9.51%, 83.55%). This could mainly be attributed to the fact that reed emerges from shallow water or low wetlands and exerts a better effect in restoring saline land, while the reed root system exerts a stabilizing effect on water and soil, which would enable effective maintenance of the functional area in JMNNR. Moreover, the reed wetland provides a habitat for waterfowl to breed and feed, thereby restoring the ecological functions of the wetland in the reserve. Furthermore, the upper and lower limits of the ESVs of vegetation carbon sequestration and oxygen release were also observed to have increased, reaching the values of (5.51%, 77.21%) and (5.54%, 77.25%), respectively. Both vegetation carbon sequestration and oxygen release are indicators of regulatory services. This increase, therefore, reflected that the ITSP model constructed in the present study could regulate the ecological and environmental functions and enhance the project benefits. The upper limits of the remaining indicator were also observed to have increased compared to the project recommendation ranging from 17.33% to 66.77%, while they had been reduced from the project recommendation ranging from −20.00% to −1.26%. After the optimization of the ITSP model simulation results, the ESV range was extended from a specific value to an interval, thereby increasing the flexibility of decision making and rendering it further convenient for the decision-makers to adapt to the actual project situation and thus develop further effective and efficient project operation and management solutions.

### 4.2. Analysis of the Changes in the Functional Area of the Ecological Water Replenishment Project in JMNNR Using the ITSP Method

**Figure 3:**
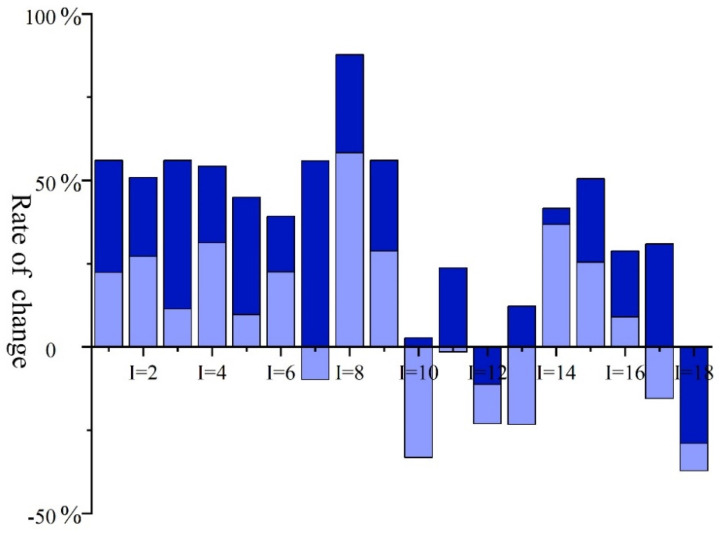
Changes in the respective areas of different functional areas of the lake in the protected area prior to and after model optimization compared to the project recommendation.

Ecological recharge is one of the most direct and effective approaches to reconstruct wetland ecosystems and restore wetland functions [25,26], which is conducive to solving the problem of shrinking wetland areas and effectively restoring the wetland areas. Table 2 shows the area value in the recommended project solution as well as the area results obtained using the ITSP method for the functional area of each lake in the reserve. Figure 3 depicts the changes in the functional area of each lake in JMNNR compared to the recommended project solution values after optimization using the ITSP model. The changes in the functional areas of the lakes in JMNNR compared to the recommended project solutions are presented in Figure 3.

The result values are presented as intervals. Compared to the one value in the project recommendation, the interval range facilitates the decision-makers in making better decisions suited to the actual working conditions. As visible in Table 2, after the ITSP model simulation, the functional area of the Zhushanpao continued to remain the largest, with the values of (7.04, 9.92) × 10^4^ hm^2^, (7.64, 12.37) × 10^4^ hm^2^, and (8.79, 13.82) × 10^4^ hm^2^ in dry, flat, and abundant water years, respectively. This was followed by Haernaopao, with the values of (6.39, 9.27) × 10^4^ hm^2^, (7.54, 12.11) × 10^4^ hm^2^, and (9.72, 13.23) × 10^4^ hm^2^ in dry, flat, and abundant water years, respectively. This was consistent with the project scheme ranking. The remaining functional area of the lake remained unchanged largely, indicating that after optimization using the ITSP method, the functional area considers the original lake division area and does not pursue the area enhancement effect. As the water period progressed, the area of the functional region increased in the order of dry, flat, and high water years. This is because the lowest level of water available in the dry years rendered it further difficult to restore the area of the functional region, while the high level of water available in the high water years rendered it convenient to restore the area of the functional region due to the flexibility in water allocation.

When the above results are analyzed in combination with Figure 3, it is visible that after optimization using the ITSP method, the area of each lake functional region had changed compared to the project recommendation. Among all lakes, the fire-burning lake presented the largest increase in both upper and lower limits of the optimized functional area compared to the project recommendation ((58.36%, 87.77%)). This was followed by Yuanbaotupao ((22.38%, 56.00%)), Ulanzhaopao ((27.19%, 50.86%)), Momogepao ((11.39%, 56.00%)), Etoupao ((31.22%, 54.24%)), Taipingshanpao ((9.68%, 44.91%)), Datunpao ((22.49%, 39.26%)), Wobupao ((28.82%, 56.00%)), Wujiazi Reservoir ((36.81%, 41.65%)), Qunying Reservoir ((25.40%, 50.55%)), and Yinghoutaipao ((9.06%, 28.80%)). The optimized upper and lower limits of the functional area were increased compared to the project recommendation, indicating that the optimized simulation of the ITSP model focused on restoring the functional area of these lakes. The lower limit of the optimized functional area of Gaomianpao ((−9.82%, 55.82%)), Zhushanpao ((−33.19%, 2.77%)), Houbutaipao ((−1.48%, 23.74%)), Haernaopao ((−23.31%, 12.23%)), and Nashitupao ((−15.46%, 30.94%)) was lower than that of the project recommendation. The lower and upper limits of the optimized functional areas of the Momoge Nature Reserve ((−15.46%, 30.94%)) were lower and higher, respectively, compared to the limits in the project recommendation, which indicated that the restoration of the functional areas of these lakes was selective and could be adjusted selectively by considering the functional areas in the ITSP as a whole. The upper and lower limits of the optimized functional area of Shaolipao ((−23.06%, −11.29%)) and Baoshanpao ((−37.17%, −28.95%)) were lower than the recommended project solution in terms of the increase in the functional area, which was because the model considered, in general, the overall lake functional area of JMNNR and could be adjusted as required, thereby causing increased overall lake functional area of JMNNR after the model simulation.

### 4.3. Analysis of the NSC of the Ecological Replenishment Project in JMNNR Using the ITSP Method

As an important ecosystem type for carbon sinks [27], wetlands represent an important component of the global carbon cycle system, because of which the wetland carbon sinks are considered highly valuable for research [28,29]. In the present study, an ecological replenishment model of JMNNR was constructed using the ITSP method to decipher the carbon sink role of the wetlands and optimize it for the important constraint of NSC. The simulation results for the NSC obtained using the ITSP model are presented in Table 3.

As visible in Table 3, the top five NSCs after optimization using the ITSP model were Zhushanpao ((7399.34, 15,742.08) × 10^4^ t), Haernaopao ((5716.78, 11,409.77) × 10^4^ t), Etoupao ((4625.40, 7427.34) × 10^4^ t), Gaomianpao ((3411.57, 6298.56) × 10^4^ t), and Yuanbaotupao ((3995.45, 6033.06) × 10^4^ t) (in flat water years), which was consistent with the ranking in the recommendation scheme. The ranking of the remaining lakes in terms of NSC remained largely unchanged. This result indicated that the model was optimized on the premise of completely considering the NCS capacity of the original lakes. The project recommendation scheme provides a single recommendation value. In contrast, in the actual project implementation process, the NSC has uncertainty and, therefore, in the present study, the results were expressed in interval form to characterize the NSC, completely considering its uncertainty and optimizing the decision scheme.

After optimization using the ITSP model, the upper and lower limits of the NSC for each lake had changed compared to the project recommendation. The upper and lower limits of NSC for the fire lake were the largest compared to the project recommendation, by up to (68.30%, 151.70%). This was because after the optimization using the ITSP model, the planning area of the functional region of the fire-burning lake increased the most, and the area with the wetland plants and having a carbon sink function, such as reeds, increased. This increased the NSC capacity of this lake. The lower limits of the NCS of Yuanbaotupao, Ulanzhaopao, Etoupao, Wobupao, Qunying Reservoir, and Yingtaihoupao were lower while their upper limits were higher than the project recommendation values, indicating that different types of functional areas could be selectively divided in these lakes to achieve the effect of controlling the NCS capacity. Both upper and lower limits of the optimized NSC in Datunpao were lower than the project recommendation, which indicated an overall consideration of the NSC capacity of JMNNR, the overall increase in the reserve’s NSC capacity. and a significant effect of the ecological function reconstruction.

### 4.4. Practical Assessment of the ITSP Model

The ITSP model constructed in this paper has wide application prospects, not only for JMMNR but also for wetland replenishment projects in other regions. When applying the model, the local characteristics should be considered and the influencing factors should be adjusted. When applying the ITSP model, control units and planning areas should be redefined according to the size of the planning area, a suitable planning cycle should be determined, the types of ecological values and indicators to be considered should be set according to the local industrial structure, and the ITSP model should be established in line with the actual local situation to provide decision-makers with a decision solution.

## 5. Conclusions

On the premise of restoring the ecological environment of the reserve, in order to maximize economic benefits, the current study involved the development of an eco-logical water replenishment model for JMNNR using the ITSP method, which aimed at the wetland water shortage, and took into account uncertainties in the ecological replenishment process and characterized simulation results as interval values, thereby broadening the scope of decision making. After optimization using the ITSP model, the ESV, functional area, and the NSC of JMNNR improved significantly, indicating that the ITSP model constructed in the present study could effectively restore the ecological functional area in the reserve and improve the ecological function of the wetland while also ensuring the economic and project benefits. Meanwhile, the ITSP model characterized the decision-making scheme in the form of intervals, which eliminated the limitation of a single decision value in the recommendation project scheme and increased flexibility in the decision-making process, both of which are conducive to decision-makers when incorporating adjustments according to the actual project profile and preparing a further practical decision-making scheme. As a practical model, the ITSP model constructed in this paper is equally applicable to wetland replenishment projects in other areas and should be used with reasonable delineation of regional control units and planning periods, taking into account the scale of the planning area and the actual regional profile, model parameters are established based on local conditions to build a water refill model suitable for the region, so as to obtain a water refill solution suitable for the region. In the process of replenishing wetlands, we should pay attention to the common development of ecology and economy. The model constructed in this paper focuses on economic development. Liao et al. (22] focused on restoring the suitable habitat area of rare birds. This paper provides another scheme for decision-makers from the perspective of economic development, which is beneficial for decision-makers to comprehensively consider the actual situation of wetland water supply. The ITSP model constructed in this paper is based on the actual situation of JMNNR, and the study scale is small, and there are no social problems such as population migration, large-scale land use change, climate change, etc. The model constraints have limitations, therefore, when decision-makers in other regions use this model for engineering decisions, they should take into account the local regional profile, but also fully consider the local topographic conditions, social factors, population migration, etc., and make relevant adjustments to the model constraints. The ITSP model constructed in the present study could also be used as a theoretical guide for conducting wetland ecological replenishment in other areas by adjusting the model to suit the local conditions during the actual implementation of the project, which would enable preparing a decision-making scheme closer to the ideal scheme.

## Figures and Tables

**Table 1:** Estimated ESV of JMNNR under the ecological service function system (×10^6^ yuan).

Ecological Service Function System	Project Recommendation	ITSP Model Optimization Results		
		*h* = *1*	*h* = *2*	*h* = *3*
Fish	78.88	(63.10, 90.71)	(63.10, 90.71)	(63.10, 99.91)
Crab	234.96	(187.97, 270.20)	(187.97, 270.20)	(187.97, 286.65)
Reeds	22.32	(23.56, 41.73)	(24.78, 41.73)	(24.98, 39.32)
Scirpus triqueter	27.00	(21.60, 36.69)	(24.78, 44.12)	(29.77, 48.37)
Water supply	125.56	(100.45, 144.40)	(100.45, 144.40)	(100.45, 155.14)
Vegetation carbon sequestration	6.92	(6.85, 12.02)	(7.34, 12.51)	(7.71, 12.24)
Oxygen release	63.73	(63.11, 110.83)	(67.62, 115.31)	(71.06, 112.75)
Food storage	412.82	(338.88, 515.08)	(349.78, 536.25)	(364.31, 570.58)
Cooling and humidifying	307.77	(268.82, 459.72)	(297.40, 515.23)	(335.51, 538.93)
Plant adsorption	308.42	(270.77, 467.14)	(301.15, 526.16)	(341.67, 549.75)
Pollution absorption capacity	446.52	(392.00, 676.30)	(435.99, 761.75)	(494.65, 795.90)
Biological Habitat	76.51	(65.46, 107.89)	(70.84, 118.33)	(78.01, 124.38)
Conservation of rare waterfowl	307.07	(262.72, 433.00)	(284.30, 474.92)	(313.08, 499.20)
Soil conservation	265.15	(229.90, 388.13)	(252.38, 431.80)	(282.36, 452.41)
Nutrient circulation	20.75	(17.99, 30.40)	(19.76, 33.84)	(22.12, 35.45)
Research and education	15.92	(13.62, 22.45)	(14.74, 24.62)	(16.23, 25.88)
Leisure and tourism	114.51	(97.97, 161.47)	(106.02, 177.10)	(116.75, 186.16)
Cityscape	62.65	(53.60, 88.34)	(58.00, 96.89)	(63.87, 101.84)
Natural landscapes	25.16	(21.53, 35.48)	(23.30, 38.91)	(25.65, 40.90)

**Table 2:** Changes in the functional area of each lake in the nature reserve prior to and after the ITSP model optimization (×10^4^ hm^2^).

Lake	Project Recommendation	ITSP Model OPTIMIZATION Results		
		*h* = *1*	*h* = *2*	*h* = *3*
Yuanbaotupao	3.58	(4.28, 5.58)	(3.93, 5.58)	(4.93, 5.58)
Wulanzhaopao	1.74	(2.43, 2.65)	(1.80, 2.54)	(2.41, 2.69)
Momogepao	3.29	(3.38, 5.13)	(3.81, 5.13)	(3.81, 5.13)
Etoupao	3.80	(4.57, 5.79)	(5.02, 5.86)	(5.37, 5.93)
Taipingshanpao	1.26	(1.23, 1.56)	(1.44, 1.96)	(1.47, 1.96)
Datunpao	1.13	(1.42, 1.57)	(1.33, 1.57)	(1.41, 1.57)
Gaomianpao	3.79	(3.03, 5.89)	(3.80, 5.91)	(3.42, 5.91)
Huoshaopao	0.48	(0.69, 0.75)	(0.69, 0.98)	(0.90, 0.98)
Wobupao	0.92	(1.02, 1.44)	(1.31, 1.44)	(1.23, 1.44)
Zhushanpao	11.71	(7.04, 9.92)	(7.64, 12.37)	(8.79, 13.82)
Houbutaipao	3.79	(3.36, 4.64)	(3.92, 4.64)	(3.92, 4.79)
Shaolipao	2.09	(1.49, 1.86)	(1.60, 1.84)	(1.73, 1.87)
Haernaopao	10.28	(6.39, 9.27)	(7.54, 12.11)	(9.72, 13.23)
Wujiazi Reservoir	0.36	(0.51, 0.56)	(0.51, 0.40)	(0.45, 0.56)
Qunying Reservoir	1.20	(1.64, 1.81)	(1.64, 1.81)	(1.23, 1.81)
Yinghoutaipao	0.20	(0.27, 0.29)	(0.25, 0.19)	(0.13, 0.29)
Nashitupao	1.00	(0.82, 1.14)	(0.82, 1.38)	(0.89, 1.41)
Baoshanpao	0.73	(0.36, 0.47)	(0.47, 0.47)	(0.54, 0.61)

**Table 3:** NSC in each lake in JMNNR (10^4^ t).

Lake	Project Recommendation	ITSP Model Optimization Results			Rate of Change
		*h* = *1*	*h* = *2*	*h* = *3*	
Yuanbaotupao	3104.47	(2483.58, 6033.06)	(3995.45, 6033.06)	(3947.37, 6033.06)	(11.95%, 94.33%)
Wulanzhaopao	1517.56	(2099.06, 3369.02)	(1233.13, 3224.13)	(2230.14, 3414.51)	(22.18%, 119.82%)
Momogepao	2844.29	(2275.43, 5546.37)	(2534.16, 5472.72)	(2534.16, 5472.72)	(−13.94%, 93.27%)
Etoupao	3301.04	(4836.73, 7331.59)	(4625.40, 7427.34)	(2673.37, 3827.86)	(22.54%, 87.69%)
Taipingshanpao	1091.18	(872.94, 2056.01)	(927.09, 2127.80)	(1191.69, 2127.80)	(−8.61%, 92.81%)
Datunpao	988.59	(820.70, 909.50)	(820.70, 909.50)	(831.73, 909.50)	(−16.61%, −8.00%)
Gaomianpao	3273.54	(2618.83, 6298.56)	(3411.57, 6298.56)	(3592.81, 6298.56)	(−2.01%, 92.41%)
Huoshaopao	426.38	(717.60, 1073.22)	(717.60, 1073.22)	(717.60, 1073.22)	(68.30%, 151.70%)
Wobupao	794.89	(1023.02, 1529.44)	(1019.34, 1529.44)	(1022.46, 1529.44)	(28.52%, 92.41%)
Zhushanpao	9249.17	(7399.34, 14,633.72)	(7399.34, 15,742.08)	(8470.54, 16,240.82)	(−16.14%, 68.00%)
Houbutaipao	2073.14	(1658.51, 3542.77)	(1804.90, 3542.77)	(1971.04, 3731.65)	(−12.62%, 73.93%)
Shaolipao	1145.5	(916.40, 1786.87)	(943.91, 1759.76)	(1069.05, 1795.04)	(−14.76%, 55.44%)
Haernaopao	6461.45	(5291.13, 10,433.41)	(5716.78, 11,409.77)	(6280.53, 11,630.61)	(−10.81%, 72.68%)
Wujiazi Reservoir	153.05	(122.44, 390.28)	(122.44, 390.28)	(200.14, 390.28)	(−3.08%, 155.00%)
Qunying Reservoir	141.57	(113.26, 297.30)	(187.30, 297.30)	(187.30, 297.30)	(14.87%, 110.00%)
Yinghoutaipao	11.48	(19.32, 29.27)	(9.18, 29.27)	(9.18, 29.27)	(9.43%, 155.00%)
Nashitupao	580.37	(480.42, 1039.50)	(564.12, 1044.67)	(564.12, 1044.67)	(−7.61%, 79.70%)
Baoshanpao	842.22	(673.78, 1012.51)	(673.78, 1012.51)	(673.78, 1309.48)	(−20.00%, 31.97%)

## Data Availability

The data presented in this study are available contained within the article.
